# Supplementary material for: Factors Influencing the Difficulty and Need for External Help during Laparoscopic Appendectomy: Analysis of 485 Procedures from the Resident-1 Multicentre Trial
Source: J Pers Med. 2022 Nov 15;12(11):1904. doi: 10.3390/jpm12111904 (PMC9697147; doi:10.3390/jpm12111904)
Supplement: Supplementary file 1 [file jpm-12-01904-s001.zip › Supplementary material S1.pdf]

Supplementary material S1.

Resident-1 Research Group.

All members of the collaborative are listed in alphabetical order and should be included as PubMed citable co-authors. ORCID id are reported where available.

Name	Surname	Email	ORCID
Giulia Arianna	Abruzzese	Giuliarianna.abruzzese@unimi.it	
Francesca	Albanesi	Francesca.albanesi@unimi.it	0000-0002-1898-1367
Michele	Altomare	Michele.altomare@ospedaleniguarda.it	0000-0002-3879-2678
Erika	Andreatta	Erika.andreatta87@gmail.com	0000-0001-5130-6710
Ludovica	Baldari	Ludovica.baldari@gmail.com	0000-0002-7029-8485
Laura	Benuzzi	Laura.benuzzi@unimi.it	0000-0003-1542-2807
Emanuele	Bevilaqua	Emanuele.bevilaqua@unimi.it	0000-0002-5998-8514
Alessandro Michele	Bonomi	Alessandro.bonomi@unimi.it	0000-0002-7248-197X
Greta	Brachetti	Greta.brachetti@unimi.it	
Giulia	Cannavale	Giulia.cannavale@gmail.com	
Andrea Piero	Chierici	Andreapiero.chierici@gmail.com	0000-0003-1475-4500
		Stefanopiero.cioffi@ospedaleniguarda.it	0000-0002-5453-8901
Riccardo	Cirelli	Riccardo.cirelli@unimi.it	0000-0003-0925-507X
Gaia	Colletti	Gaia.colletti@unimi.it	0000-0001-9859-836X
Vera	D'abrosca	Vera.dabrosca@unimi.it	
Piergiorgio	Danelli	Piergiorgio.danelli@unimi.it	0000-0003-1461-9835
Luca	Del Prete	Luca.delprete@policlinico.mi.it	0000-0003-0268-3266
Francesco	Di Capua	Francesc.dicapua@gmail.com	0000-0001-6636-8172
Francesca	Di Vittorio	Divittorio.francesca@hsr.it	0000-0002-8978-3798
Davide	Ferrari	Davide.ferrari@unimi.it	
Luca	Ferrario	Luca.ferrario1@unimi.it	0000-0002-3652-3255
Laura	Fiore	Laurafiore@msn.com	0000-0002-7360-3625
Colomba	Frattaruolo	Colomba.frattaruolo@unimi.it	0000-0001-6165-2641
Caterina	Froiio	Caterina.froiio@unimi.it	0000-0003-1018-3691
Ludovica	Gibelli	Ludovica.gibelli@unimi.it	0000-0002-1230-0057
Irene	Giusti	Irene.giusti@unimi.it	0000-0002-6930-3144
Ugo	Giustizieri	Ugo.giustizieri@unimi.it	0000-0003-3100-4496
Samuele	Grandi	Samuele.grandi@unimi.it	0000-0002-9257-0240
Stefano	Granieri	Steff.granieri@gmail.com	0000-0001-9167-3400
Giulio	Iacob	Giulio.iacob@unimi.it	0000-0002-8929-1304
Alessia	Kersik	Alessia.kersik@unimi.it	0000-0002-2888-9572
Pietro	Lombardi	Pietro.lombardi@humanitas.it	0000-0002-2633-7977
Marco	Longhi	Marco.longhi@asst-lodi.it	0000-0001-9581-4450
Leonardo	Lorusso	Leonardo.lorusso@unimi.it	0000-0001-9581-4450

Michele	Manara		
Elena	Manzo	Elena.manzo@unimi.it	0000-0001-6558-5431
Jacopo Nicolo	Marin	Jacopo.marin@unimi.it	0000-0001-6951-6695
Marianna	Maspero	Marianna.maspero@unimi.it	0000-0002-7589-4489
Valentina	Messina	Valentina.messima@unimi.it	0000-0003-0127-4161
Pamela	Milito	Pamela.milito@unimi.it	0000-0002-5926-3943
Mattia	Molteni	Molteni.mattia@hsr.it	0000-0003-1471-439X
Eleonora	Monti	Eleonora.monti44@gmail.com	0000-0001-8178-7789
Vincenzo	Nicastro	Vincenzo.nicastro@unimi.it	0000-0002-0893-9640
Giorgio	Novelli	Giorgio.novelli@unimi.it	0000-0001-9506-0871
Sissi	Paleino	Sissi.paleino@asst-brianza.it	0000-0001-7315-763X
Silvia	Pavesi	Silvia.pavesi@unimi.it	
Carolina	Perali	Carolina.perali@unimi.it	
Isabella	Pezzoli	Isabella.pezzoli@unimi.it	0000-0003-0498-8258
Roberta	Ragozzino	Roberta.ragozzino@ospedaleniguarda.it	0000-0002-0723-397X
Giuliano	Santolamazza	Giuliano.santolamazza@unimi.it	0000-0002-0656-4849
Luca	Scaravilli	Luca.scaravilli@unimi.it	0000-0002-1326-1862
Andrea	Spota	Andrea.spota@ospedaleniguarda.it	0000-0003-2288-361X
Gilda	Tornatore	Gilda.tornatore@gmail.com	0000-0001-7947-4572
Francesco	Toti	Francesco.toti@unimi.it	0000-0001-9105-4431
Vincenzo	Tripodi	Vincenzo.tripodi@unimi.it	0000-0001-6611-6477
Elisa	Vaterlini	Elisa.vaterlini@libero.it	
Barbara	Vignati	Barbara.vignati@unimi.it	0000-0003-0454-2432
