# Supplementary material for: Factors Influencing the Difficulty and Need for External Help during Laparoscopic Appendectomy: Analysis of 485 Procedures from the Resident-1 Multicentre Trial
Source: J Pers Med. 2022 Nov 15;12(11):1904. doi: 10.3390/jpm12111904 (PMC9697147; doi:10.3390/jpm12111904)
Supplement: Supplementary file 1 [file jpm-12-01904-s001.zip › Supplementary material S2.pdf]

**Supplementary material S2.**

Participating centers

1. ASST GOM Niguarda, Milan, Italy
2. ASST Rhodense, Ospedale di Rho, Rho, Italy
3. ASST Santi Paolo e Carlo, Ospedale San Carlo Borromeo, Milan, Italy
4. ASST Fatebenefratelli-Sacco, Ospedale Fatebenefratelli, Milan, Italy
5. ASST Santi Paolo e Carlo, Ospedale San Paolo, Milan, Italy
6. Ospedale Bolognini, Seriate, Italy
7. ASST Valle Olona, Ospedale di Busto Arsizio, Busto Arsizio, Italy
8. Ospedale “San Leopoldo Mandic” di Merate, Merate, Italy
9. Policlinico di Milano Ospedale Maggiore, Fondazione IRCCS Ca' Granda, Milan, Italy
10. ASST Milanese Ovest, Ospedale Giuseppe Fornaroli, Magenta, Italy
11. ASST Melegnano Martesana, Ospedale Vizzolo Predabissi, Italy
12. ASST Nord Milano, Ospedale di Sesto San Giovanni, Sesto San Giovanni Italy
13. IRCCS Multimedica, Sesto San Giovanni, Sesto San Giovanni, Italy
14. ASST Valle Olona, Ospedale di Saronno, Saronno, Italy
15. ASST Rhodense, Ospedale di Garbagnate Milanese, Garbagnate Milanese, Italy
16. Ospedale “Sacra Famiglia” Fatebenefratelli di Erba, Erba Italy
17. ASST Brianza, Ospedale di Vimercate, Vimercate, Italy
18. IRCCS Policlinico San Donato, San Donato Milanese, Italy
19. Policlinico San Marco di Zingonia, Osio Sotto, Italy
20. Ospedale Valduce, Como, Italy
21. ASST Fatebenefratelli-Sacco, Ospedale Sacco, Milan, Italy
